# Field-induced magnetic phases in a qubit Penrose quasicrystal

**DOI:** 10.1126/sciadv.adf6631

**Published:** 2023-03-17

**Authors:** Alejandro Lopez-Bezanilla, Cristiano Nisoli

**Affiliations:** Theoretical Division, Los Alamos National Laboratory, Los Alamos, NM 87545, USA.

## Abstract

Unveiling the fundamental dynamics of naturally or artificially formed magnetic quasicrystals in the presence of an external magnetic field remains a difficult problem that may have implications for the design of information processing devices. By embedding a qubit magnetic Penrose quasicrystal into a quantum annealer, we were able to reproduce the formation of magnetic phases driven by specific physical parameter selections, allowing us to distinguish a wide range of frustrated magnetic configurations at the single-spin scale. In our experiments, we observe some spins dynamically activate, while others remain static, all within an average magnetization space defined by competing structural and magnetic degrees of freedom. Static spin structure factors reveal ferromagnetic and ferrimagnetic modulations that are compatible with a variety of spin textures. This research demonstrates that introducing structural aperiodicity in magnetic devices that exploit spin degeneracy in a single, richly intraconnected finite object can enable the engineering of quantum states in both the effective low-temperature and thermally excited regimes.

## Teaser

Aperiodic lattices hosting rich magnetic patterns provide design options for information processing devices.

## INTRODUCTION

Miniaturization of storage devices motivates the search for magnetic domains modifiable with external fields and able to pack in denser arrangements. Candidates for ultrahigh-density data storage include domain walls (*1*), antidots (*2*, *3*), and magnetic pseudo-particles such as skyrmions (*4*) and vortices (*5*, *6*) where cooperative interactions between the constituent elements allow for small-size stable configurations. Furthermore, the application of an external field may alter the energy landscape of the magnetic domain and results in a polarity switch between two states (*7*, *8*), which translates into a bit of information.

An interesting yet unanswered question is whether such an effect can be achieved with magnetic units arranged in an aperiodic fashion so that magnetic phases able to evolve in regimes crucial to magnetic switching applications can be created. Aperiodic lattices such as quasicrystals (*9*, *10*) have a rich node connectivity that may vary greatly from one vertex to the next, resulting in spatial variations of the local energy density. Therefore, multiple magnetic states are expected to exist in a quasicrystal depending on the local magnetization at a specific vertex (*11*). This could help to mitigate the effects of supermagnetism (*12*–*14*), which occurs when thermal fluctuations cause a spontaneous reversal of magnetization in small-volume components. Maximizing the energy barriers separating equilibrium states is a usual approach to circumvent the detrimental effect of random deviations of magnetization from its average value. An approach based on the interplay between an aperiodic topology, magnetic strength, and external fields is proposed here to harness energy barriers and temperature-induced stochastic magnetization dynamics, which may pave the way to a better control of spin textures while offering the prospect of encoding more than one bit of information in a single object.

Here, we design and build a customized quasicrystal in a quantum annealer (QA) to experimentally investigate a mechanism capable of generating multiple magnetic phases in an aperiodic, richly intraconnected physical object. We show that coupling each qubit to a modulating longitudinal magnetic field allows us to manipulate the quasicrystal magnetization and the gradual emergence of stable magnetic textures. The fabrication of the logic quasicrystal is compatible with conventional qubit lattice embedding methods on QAs, making this quantum platform an effective tool for probing the expected interaction mechanism in an actual material. The integrated control of creating, annihilating, and morphing of magnetic arrangements is realized simultaneously in multiple regions of one quantum processing unit, facilitating the generation of millions of low-energy magnetic state realizations.

A quasicrystal exhibits defined diffraction patterns but symmetries forbidden in periodic crystals. Considered as a result of projections of higher-dimensional lattices, these mathematical objects find their incarnation on various materials, from Al-Mn alloys (*15*) to byproducts of nuclear device detonation (*16*). Two-dimensional quasicrystals include the tiles proposed by Penrose (*17*), a set of discrete and countably infinite number of nodes in locally similar structures. Since their initial experimental description (*15*), quasicrystals have been the subject of hundreds of studies. Prior explorations of magnetic correlations were around the pervasively observed spin glass–like freezing behavior (*18*–*20*), until the recent report of long-range magnetic order in an icosahedral quasicrystal (*21*). Monte Carlo computational methods estimated the critical temperatures of Penrose lattice models (*22*, *23*) and concluded that the system belongs to the same universality class as the Ising model on regular Bravais lattices (*24*–*26*).

Concerns about the difficulty of creating an atomic arrangement that matches the Penrose tiling rules prompted the development of alternative models that allowed the effects of quasicrystallinity to be observed using tailor-designed structures. On the basis of nanofabrication techniques (*3*, *27*–*30*), 10-fold rotational symmetry photonic (*31*, *32*), magnonic (*33*), graphene-based (*34*), and molecular quasicrystals (*28*, *35*) have been experimentally designed. In macroscopic artificial magnetic quasicrystals (*36*, *37*), the magnetic configuration follows the ice rule of square and kagome tessellations to minimize the total energy, i.e., the macrospins at each vertex point in and out of a regular polygon to reach a lower-energy state. These models were implemented in a QA to simulate the physics of artificial spin ices at the single-spin level, allowing for the observation of magnetic monopoles (*38*) and fractional excitations (*39*).

## RESULTS AND DISCUSSION

**Fig. 1.**
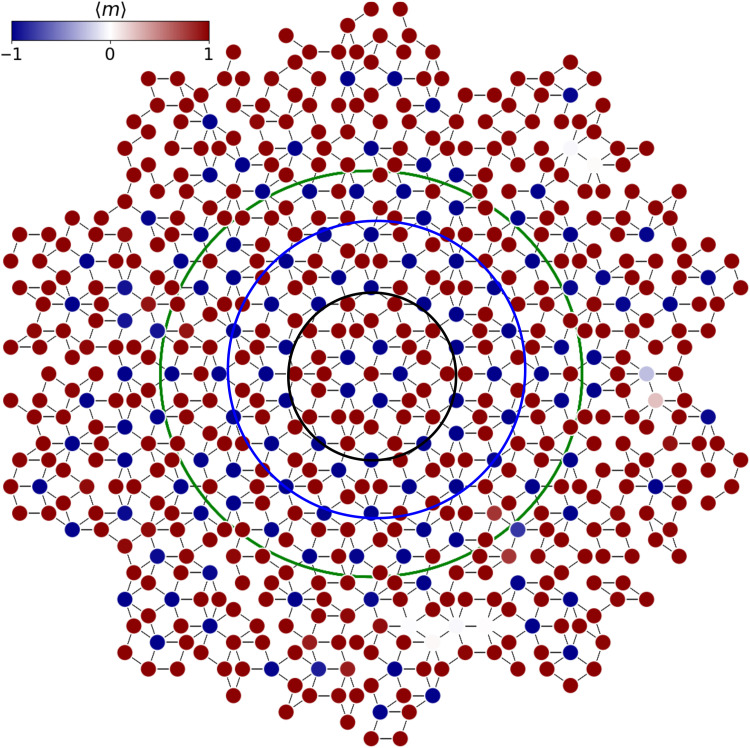
Penrose quasicrystal. Representation of the largest and smaller Penrose P3 quasicrystals embedded in the QA. Green, blue, and black lines that delimit the three defect-free approximants studied here are composed of single-edge length fat and thin rhomb tiles in which each vertex represents a qubit that is connected from 2 to 7 other qubits. Lines joining vertices represent an AFM isotropic coupling between qubits. Blue-to-red gradient denote spin orientation within a zero-field AFM Ising model. Quasicrystal lattices are bipartite in the absence of defects: Blue and red sites alternate at first neighbors.

A programmable QA is used to mold a broad range of collective magnetic states in a finite-sized Penrose P3 lattice. Experiments on superconducting qubits were conducted to analyze the conditions leading to frustrated low-energy configurations of a nearest-neighbor antiferromagnetic (AFM) spin-1/2 Ising model (*40*, *41*). Figure 1 displays one of the largest, although irregular, P3 approximants (600 qubits) compatible with the Pegasus QA topology that we succeeded to embed. We focus on regular, fivefold symmetrical, and isotropic P3 topologies where the connectivity of *n*-fold (*n* = 2…7) coordinated vertices enables a rich breadth of homogeneous, well-controlled magnetic field-driven phases unusual in regular crystals and thin-layered ferromagnetic materials (*42*, *43*).

**Fig. 2.**
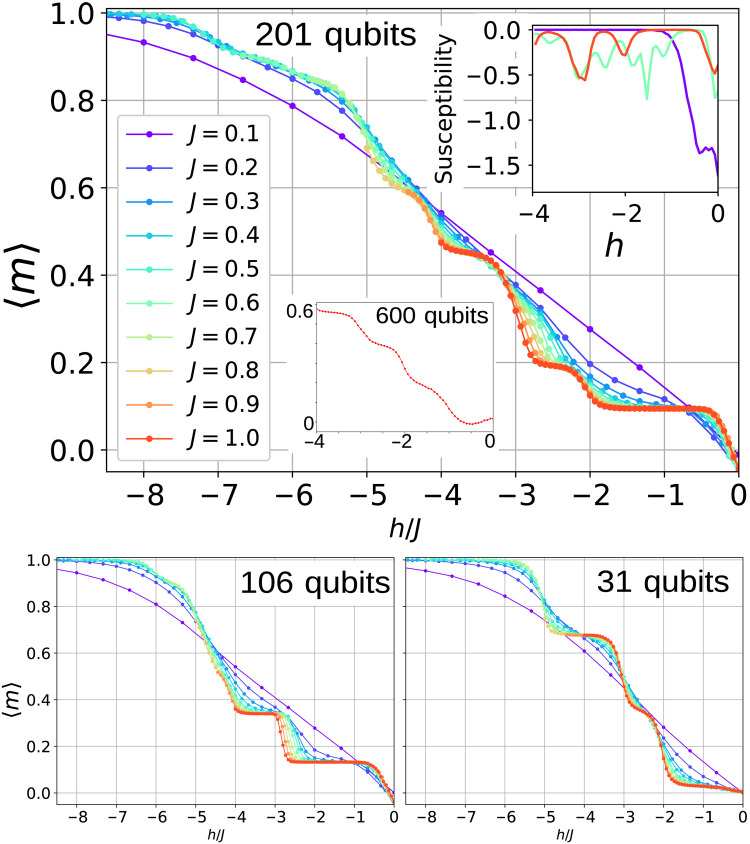
Isothermal out-of-plane average magnetization. 〈*m*〉 of the Penrose lattice for increasing longitudinal field *h* strengths and for four different lattice sizes: 600, 201, 106, and 31 qubits as delimited in Fig. 1. A net 〈*m*〉 at *h* = 0 is due to the imbalance in the bipartite lattice site number. The oscillatory behavior of the susceptibility (top right inset) is a consequence of the fluctuations of 〈*m*〉 for each *J* = 0.1,0.6, and 1.0. Plateaus of the irregular 600-qubit lattice blur as a result of the defects (bottom center inset).

Figure 1 shows a spin density field of the 600-qubit defect-rich quasicrystal in the absence of an external magnetic field (*h* = 0). While the presence of vacant vertices precludes the formation of a single ground state, bipartite fivefold symmetrical AFM Penrose lattices in the low-thermal fluctuation regime exhibit degeneracy, beyond the time-inversion symmetry. This translates into an alternating distribution of spins, where a spin-up (+1) classical state is always connected at first neighbors to a spin-down (−1) classical state. The application of a longitudinal field *h* introduces an external degree of freedom that couples to the internal field and increases the lattice magnetization *m*. Figure 2 shows the evolution of 〈*m*〉 = 〈 ∣ *n*_+1_ − *n*_−1_ ∣ 〉 (*n*_+1_ and *n*_−1_ are the number of magnetic moments in +1 and −1 orientations), for an increasing ∣*h*∣, and a series of AFM *J* terms. While the average magnetic moment of some qubits changes in one direction, others move in the opposite, giving to the transition between plateaus a progressive rather than a hard-stepwise shape. Only the imperfect connectivity of the largest quasicrystal makes 〈*m*〉 adopt a quasi-linear evolution. The size and boundary shape of the quasicrystal contribute to 〈*m*〉 as well, as shown in figs. S3 and S4 for two smaller symmetric quasicrystals. At high *J* values, the magnetization versus *h* curves evolve in steps that correspond to integer ratios of the field over the coupling constant. The series of curves shows the progressive data collapse in *h*/*J* as *J* intensifies. The presence of magnetization plateaus in the high coupling regime, the stepwise transition between them, and the absence of negative variation of <*m*> with field strength can be all considered as characteristics of a symmetric quasicrystal, as shown in Fig. 2. It must be noted that because *<m*> is averaged over the entire ensemble of magnetic moments, its stepwise shape can change depending on how the individual sets of qubits evolve and contribute, resulting in smoother transitions. As a result, observing the individual evolution of each set of equivalent qubits can provide additional insight into the degree of symmetry. The statistics are realized over 8 × 10^4^ collected classical state low-energy configurations for each specific combination of *J* and *h*.

The averaged magnetization steps match natural values of *h*/*J* in correspondence to the coordination of the vertices. In a first approximation, a magnetic moment with *n* neighbors in opposite direction contributing a local field of −*n* × *J* would flip if *h*/*J* < − *n*. The sharped magnetization transitions for strong coupling are clear in the susceptibility plot (Fig. 2, inset). While this simple argument is valid in a zeroth-order conceptualization, the interaction mechanism ruling the magnetic moment orientation is intricate due to the collective nature of the system, as will be shown next.

**Fig. 3.**
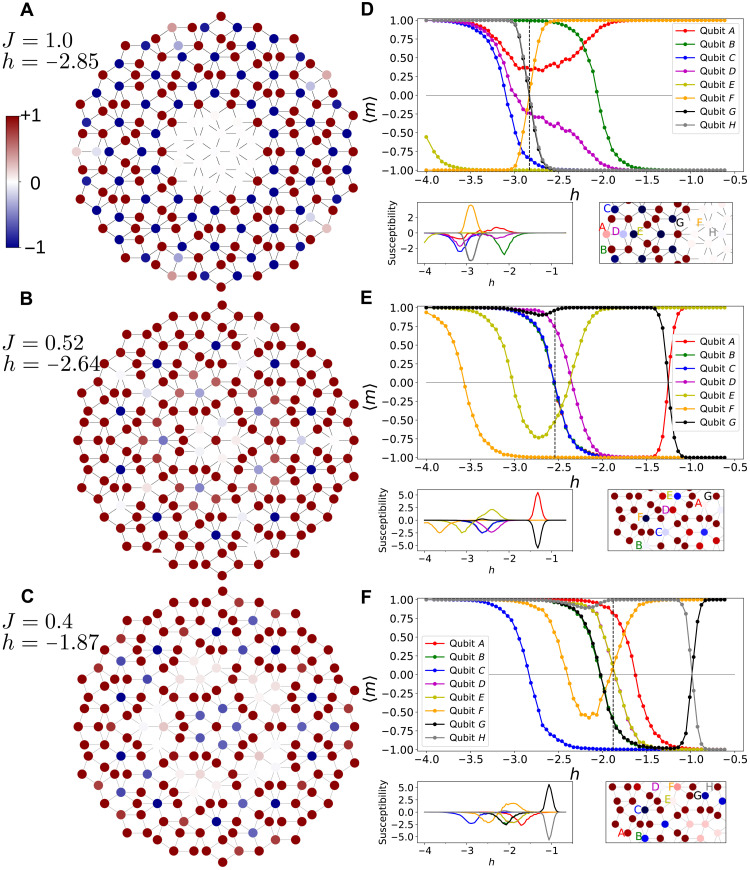
Qubit-resolved averaged magnetizations. (**A** to **C**) Averaged magnetizations of each qubit the P3 Penrose lattice under combinations of local and external magnetic parameters. Vanishing qubits correspond to frustrated magnetic states that are dynamically activated over subsequent annealing cycles. (**D** to **F**) Evolution with applied longitudinal field of the average magnetization (top) and magnetic susceptibility (bottom left) of selected individual qubits for each *J* = 1,0.52,0.4. In (D), *A* to *H* qubits belong to *C*_2_, *C*_3_, *C*_4_, *C*_5_, *C*_12_, *C*_26_, *C*_24_, and *C*_27_, respectively. In (E), green line is superimposed by blue line. Red line is superimposed by blue and green lines for *h* < − 1.5. *A* to *G* qubits belong to *C*_26_, *C*_11_, *C*_18_, *C*_21_, *C*_23_, *C*_16_, and *C*_27_, respectively. In (E), green line is partially superimposed by black line for *h* < − 1.8 and by yellow line for *h* > − 1.3. *A* to *H* qubits belong to *C*_6_, *C*_11_, *C*_13_, *C*_20_, *C*_21_, *C*_23_, *C*_26_, and *C*_27_, respectively. Vertical dashed line points out the h at which states are plotted in (A) to (C).

Figure 3 (A to C) shows a real-space visualization of the on-site–resolved average magnetization for three combinations of *J* and *h*. The probability of a magnetic moment of being oriented in a +1 or −1 state is modified by its coupling with the external field. According to 〈*m*〉 observed on each site, a low *T*_eff_ of *J* = 1.0 combined with *h* = − 2.85 leads to the dynamical activation of central qubits in *C*_25_, *C*_26_, and *C*_27_ (Fig. 3A). See Methods for a description of the notation used here for a qubit of class *n* (*C_n_*) and the definition of the effective temperature *T*_eff_. The torque imposed by *h* favors the anti-alignment and allows those magnetic moments to overcome the AFM coupling with the neighboring qubits. Initially, strongly polarized states are brought into degeneracy, and both +1 and −1 orientations own the same probability to be observed.

The variation of 〈*m*〉 for selected qubits at *J* = 1 is shown in Fig. 3D. While *B* is a twofold-coordinated atom that changes orientation under the action of a weak field (*h* = 1.5), *C* requires a stronger field (*h* = 2.5) to overcome the coupling with its three neighboring qubits. *D* is also a threefold-coordinated qubit but undergoes a more progressive magnetization change, starting short after *B* at *h* = 1.7 and with several peaks in its susceptibility (see inset). *A* keeps a positive magnetization throughout the action of the longitudinal field, but the change of its susceptibility runs parallel to that of *D*. Therefore, although *A* and *D* qubits are coupled to each other and both are threefold-coordinated, their different environments determine the evolution of their susceptibility. The threefold-coordinated qubit *D* changes its orientation at a unit of *h* higher than the twofold-neighbored qubit *B*. Both exhibit 〈*m*〉 ∼ 0 when the coupling energy with the external field is nearly that of the local field exerted by neighboring spins.

The action of an increasing ∣*h*∣ along the −*z* direction tends to flip qubits with initially 〈*m*〉 < 0. Note that only *F* qubits exhibit 〈*m*〉 > 0 at low ∣*h*∣ and flips with increasing strength of *h* to finish in a field-aligned configuration. Neighboring qubits of *F* follow the same magnetization evolution despite the fact that the different threefold (*G*) and fivefold (*H*) coordinations provide very different local energetic conditions. The crossover at *h* = − 2.87 pinpoints the dynamical suppression point, where the symmetric but opposite evolution of those qubits magnetization in the central region yields 〈*m*〉 = 0. It is worth emphasizing that all qubits exhibit an orientation at every readout, but those vanishing display 〈*m*〉 = 0 over the total time the measurements are taken.

Counterintuitively, reducing the coupling between qubits down to *J* = 0.52 and ∣*h*∣ by 10%, *C*_25_ and *C*_26_ qubits recover a strong magnetization (see Fig. 3B). With *h* not strong enough to take those threefold-coordinated magnetic moments to degeneracy, only the fivefold-coordinated spins of *C*_10_ and *C*_18_ are activated. The qubit sets of *C*_20_, *C*_21_, and *C*_23_ are also fivefold-coordinated, but their energy is only partially lifted by the longitudinal field. This cluster of spins further reduce their average magnetization upon lowering the internal coupling and the applied field. At *J* = 0.4, thermal fluctuations start inducing transitions at both sides of the energy barrier between the otherwise metastable or minima points. Although the P3 approximant is fivefold symmetric, differences in 〈*m*〉 between qubits of a same class are expected. In the high-coupling (*J* = 1) regime, magnetic moments are less affected by thermal fluctuations and only statistical deviations introduce a bias of toward non-zero values of 〈*m*〉. At low *J* values (high *T*_eff_), 〈*m*〉 is more prone to deviations because the final magnetization is subject to supermagnetic variations.

Simultaneously, the formerly activated spins recover partially their magnetization while *C*_6_ fourfold-coordinated spins continue to flip in average. Note that, in these two cases, the central qubit exhibits a robust magnetization when its first-neighbor qubits are either dynamically activated or polarized. Additional combinations of fields and coupling strength constants can create different spin textures, such as *J* = 0.6 and *h* = − 3.6, which anti-aligns all qubits except the sixfold-coordinated *C*_23_ and sevenfold-coordinated *C*_13_ qubits that are dynamically activated and aligned with the field, respectively.

The complex interplay between geometry and magnetic degrees of freedom dictates the final spin texture of the quasicrystal. Different sets of qubits with the same coordination can be activated at different applied *h*, so that the simple *h* = − *n* × *J* for 〈*m*〉 = 0 is often the exception. Although coupling is set only at first neighbors, 〈*m*〉 depends on the coordination of qubits beyond that nearest-neighbor constraint, showing in full its collective nature. Evolution of 〈*m*〉 with *h* for individual qubits coupled with both *J* = 0.52 and *J* = 0.4 is provided in Fig. 3 (E and F, respectively). The crossover that yields a similar texture to that in Fig. 3A is observed for *J* = 0.52 when *h* = − 1.25 and for *J* = 0.4 when *h* = − 0.98, namely, the higher the effective temperature (1/*J*), the lower the field to dynamically activate the central magnetic moments. Although some magnetic textures are common or similar for different sets of *J* and *h*, not all of them can be accessed by fixing *J* and tuning *h*. While at *J* = 1 all textures containing activated qubits are grouped in the range of Δ*h* = 1.1, lowering the effective temperature widens it, Δ*h* = 2.32 for *J* = 0.52, and narrows it, Δ*h* = 1.85 for *J* = 0.4. As the effective temperature increases, the field range where transitions group is in an even narrower Δ*h*. Therefore, for a given quasicrystal geometry, obtaining a precise magnetic ordering depends on both temperature and applied field. The Supplementary Materials contain animated representations of the variation of 〈*m*〉 with *h*.

By using smaller quasicrystals, we demonstrate that some features observed in the 201-site model are preserved when the size, number of components, and boundary shape are changed. A scaling analysis was performed on two 31- and 106-qubit large fivefold symmetrical P3 quasicrystals derived from the 201-site model. Figure 2 (B and C) depicts a different evolution of 〈*m*〉 based on the these two geometries and site connectivity, while figs. S3 and S4 display the individual contribution of each relevant qubit. One of the most notable findings is that the dynamical suppression of the central qubits is resilient to changes in the boundary conditions (106 large objects). Only in the smallest object (31 qubits), where those qubits are affected by their neighbors in the boundary, they keep the zero-*h*–field AFM distribution of the bipartite lattice. Figure S4 shows that this magnetic arrangement is reproduced for *J* = 0.52 and *J* = 0.4 at 〈*m*〉 = 0 for the two lines below *h* = 1.5. The suppression of the first-neighbor qubits to the central spins at *J* = 0.52 and *h* ∼ − 2.6 is preserved in all three cases, similarly for the fivefold-coordinated *C*_18_ qubits. Therefore, the central qubit remains unaltered only in the smallest quasicrystal, and its 〈*m*〉 is subject to the same variation for larger sizes, regardless the shape and behavior of the boundary. This can be further verified in the largest irregular quasicrystal (see fig. S5) where the same set of magnetic moments are dynamically suppressed under the same magnetic conditions and despite the presence of vacant sites that plague the outer areas.

Because of the long-range ordering of the structural motifs and despite that they are not topologically equivalent to a Bravais lattice, Penrose lattices own quasicrystalline diffraction characteristics (*15*). Similarly, the spin textures display a rotational symmetry inconsistent with lattice translations, but neutron diffraction patterns of quasicrystals can be defined in the likeness of those from regular crystals, a signature of the long-range order of the spin distribution (*42*, *43*). While the real-space representation of the magnetic textures imposes some limitations when it comes to observing the spin distribution, the spin structure factor *S*(*q*) allows us to distinguish the richness of the underlying spin orientations through a spatial average over the whole system. *S*(*q*) is information that can be obtained from neutron-scattering experiments, procuring a guide to unveiling the spin arrangements under each specific physical condition. The quality of our experiments is verified by plotting the static structure factor $S(\vec{q})$ defined as (*44*)

$$S(\vec{q})=\frac{1}{N_{s}}\sum_{ij}e^{-\vec{q}({\vec{r}}_{i}-{\vec{r}}_{j})}\langle {\vec{s}}_{i}\cdot {\vec{s}}_{j}\rangle$$ (1)

where *N_s_* is the number of logical spins, $\vec{q}=(q_{x},q_{y})$ is a generic two-dimensional vector of the reciprocal space lattice and ${\vec{r}}_{i}-{\vec{r}}_{j}$ is the vector between spins ${\vec{s}}_{i}$ and ${\vec{s}}_{j}$. ⟨ · ⟩ is the ground-state expectation value. $S(\vec{q})$ determines the structure of a magnetic material, including the directions the moments point out in an ordered spin arrangement and the interaction between them. Structure factors are computed at the points of magnetic degeneracy, where frustrated spin regions coexist with fixed and stable local configurations.

**Fig. 4.**
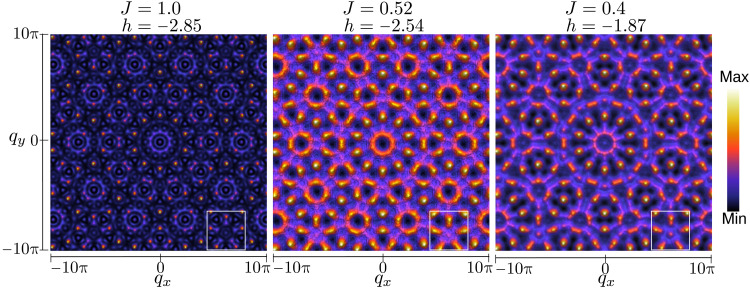
Quasicrystal structure factors. The rich variety of magnetic configurations is reflected in the structure factor diagrams of various phases. White squares point out some of the pentagonal shapes in reciprocal space denoting the fivefold symmetry of the quasicrystal.

Figure 4 displays $S(\vec{q})$ in (*q_x_*, *q_y_*) ∈ ( − 10π,10π) for the spin distributions displayed in Fig. 3 (A to C), showing evidence of the quasi-periodic long-range magnetic order. A very structured plot is obtained for the magnetic diffraction pattern at *J* = 1.0 with strong intensity spots (Bragg peaks) surrounding medium-intensity granular structures arranged circularly with 10-fold symmetry. This variety in the spot distribution and intensity is in contrast to those of *J* = 0.52 and *J* = 0.4, all exhibiting in the Fourier transforms of the spin correlation function a pervasive pentagonal symmetry.

In conclusion, the broad configuration landscape of a finite-field AFM Ising model on the Penrose P3 quasicrystal using a QA has been examined. Our approach goes beyond macroscopic experimental designs and traditional numerical algorithms of micromagnetic frameworks, enabling the study of the evolution of spin level–resolved magnetic states driven by quantum fluctuations. Experimental results on qubits have established variegated and unique spin patterns in an aperiodic topology where the interplay between local interactions, external magnetic fields, and a rich node connectivity allows us to bring the nonfrustrated state of the bipartite lattice into multiple states of degenerate magnetic moments in equilibrium with respect to field variations. Some magnetic arrangements, such as the central cluster of dynamically suppressed qubits, are nonsensitive to structural defects and uneven boundaries sufficiently far away from them. At the same time, this result reveals the importance of designing symmetric, defect-free quasicrystals to host well-defined magnetic textures over the whole object.

Consideration of the occurrence of an Ising spin liquid state is prompted by the observation of the absence of magnetic ordering in the group of interacting central spins at very low temperature in Fig. 3A. Recent reports (*45*) showed evidence of quantum coherence signatures, which prompt us to suggest additional research to demonstrate how quantum fluctuations can overcome classical degeneracy and induce new correlations toward the goal of realizing well-known or even new models of quantum spin liquids. Future research is left to undertake experiments to observe fractionalized excitations as a means of verification.

The quasicrystal structure can be viewed as a flexible substrate capable of hosting multiple magnetic textures while being resistant to thermal fluctuations for sufficiently strong coupling between magnetic moments. The QA platform’s isolated and well-controlled environment allowed us to study the effect of structure-magnetic coupling in detail in the absence of other interactions. To complement this method, it would be desirable to find an actual material to account for the effect of other constraints, such as the decoherence of spin states caused by phonon and long-range dipolar interactions. Nonetheless, the experimental evidence provided about a field-induced activated and thermally limited magnetization mechanism may serve as a benchmark for future material realizations [one–atomic layer–thick systems (*46*) and molecular frameworks (*47*)] and inspire the design of magnetic memory elements where each phase can translate into encoding a bit of information. Interactions of magnetic quasicrystals with other magnetic excitations and light are possible routes to new states of matter.

## METHODS

A high-density cluster embedding model based on a single qubit per vertex design maximizes the simultaneous utilization of 1608 qubits in a Pegasus lattice to embed up to eight 201-qubit quasicrystals (see fig. S2 and Methods for reproducibility), eliminating errors derived from broken logical qubit chains (*48*). This approach allows us to fully exercise the experimental power of the quantum platform maximizing the number of retrieved configurations in one single experiment. The Advantage4.1 quantum processing unit by D-Wave Systems (*49*) provides a venue to exploring the low-energy configuration manifold with unprecedented control over the set of parameters defining the system (*50*). Experiments were conducted on a quantum processing unit at 15.4 mK.

The quasicrystal model includes interactions between pairs of spins sitting on two contiguous vertices and defines to them a same AFM coupling, *J*. With discrete variations of the coupling strength (*J* = 0.1…1.0 in steps of 0.1), we mimic the effect of the effective temperature, which can be regarded as inversely proportional to the coupling strength between logical qubits (*T*_eff_ ∼ 1/*J*). In a system subject to high temperature, the interacting spins can be considered as decoupled, because thermal fluctuations overwhelm the energy constraint imposed by the local field. A low-temperature regime is equivalent to considering that thermal fluctuations are inefficient to induce a spin flip so that coupling between spins dominates the interaction. Therefore, a homogeneous weak coupling between individual qubits imposed throughout the system is considered as a high-temperature state. Conversely, imposing the maximum *J* between pairs of qubits mimics a state where thermal fluctuations are the lowest.

The operator governing the system dynamics is given by a classical Ising Hamiltonian whose ground state is found using quantum adiabatic optimization (*51*). The time-dependent Hamiltonian smoothly evolves from a trivial quantum superposition to a complex classical energy function

$$H(s)=J(s)\left[\sum_{i}h_{i}\sigma_{i}^{z}+\sum_{i,j}J_{ij}\sigma_{i}^{z}\sigma_{j}^{z}\right]-\text{Γ}(s)\sum_{i}\sigma_{i}^{x}$$ (2)

where *s* = *t*/*t_f_*, defined in the 0 to −1 range, is a unitless annealing parameter controlling the annealing progress; Γ(*s*) is a transverse field strength that decays to zero for *s* = 1; and 𝒥 is an Ising energy scale that increases from zero at *s* = 0. The classical Ising Hamiltonian multiplying 𝒥 is determined by one local longitudinal field *h_i_* and two local AFM exchange integral *J_ij_*. The sum over *i*, *j* runs over the set *j* of first neighbors of *i*. All pairwise coupling terms along the lattice edges are considered equal so that *J_ij_* = *J*.

We use the forward annealing technique by which the qubits are initialized in a quantum state upon application of the transverse field, 𝒥(*s* = 0) = 0. Under the effect of the quantum fluctuations, the systems explore all possible solutions. Subsequently, as the field fades out, Γ(*s* > 0) slowly ramps up the relative strength of the couplings and biases. During the anneal process, the system undergoes a phase transition from a disordered quantum paramagnetic phase to an ordered classical magnetic phase. The latter is the final state that we measure and collect as a solution of the problem, which is given by +1 and −1 states representing the final orientation of the logical spin. The system is frustrated when all constraints cannot be satisfied simultaneously, and some qubits could be in both a +1 and −1 classical configuration without affecting the total energy of the lattice. The annealing time separating *s* = 0 from *s* = 1 is 20 μs, somehow longer than the qubit coherence time (*45*), and we therefore expect open quantum dynamics in a system coupled to a thermal bath. At *T* = 15.4 mK, the unitless temperature $T/J(s)\approx \frac{1}{2}$ mid-anneals when Γ(*s*) = 𝒥(*s*) and decreases to ≤0.1 when *s* = 1.

For an insightful characterization, a set of graph automorphisms to classify related lattice sites will be used. In a defect-free lattice, each spin (except for the middle one) is related to other spins by rotational symmetry operations that can be grouped into classes containing each either 5 or 10 qubits in them. Therefore, spins of the same class are automorphic to each other and must have the same magnetization under the same physical conditions, to the extent allowed by thermal fluctuations. The group of spins in each of the 27 classes of the 201-qubit approximant is given in fig. S1. A qubit belonging to class *n* will be denoted by *C_n_*.
