# Supplementary material for: Field-induced magnetic phases in a qubit Penrose quasicrystal
Source: Sci Adv. 2023 Mar 17;9(11):eadf6631. doi: 10.1126/sciadv.adf6631 (PMC10022899; doi:10.1126/sciadv.adf6631)
Supplement: Supplementary file 1 — Figs. S1 to S5 Legends for movies S1 to S9 [file sciadv.adf6631_sm.pdf]

Science Advances
AAAS

Supplementary Materials for

Field-induced magnetic phases in a qubit Penrose quasicrystal

Alejandro Lopez-Bezanilla and Cristiano Nisoli

Corresponding author: Alejandro Lopez-Bezanilla, alejandrolb@gmail.com

Sci. Adv. **9**, eadf6631 (2023)
DOI: 10.1126/sciadv.adf6631

The PDF file includes:

Figs. S1 to S5
Legends for movies S1 to S9

Other Supplementary Material for this manuscript includes the following:

Movies S1 to S9

Supplementary materials

Figs. S1, S2, S3, S4, and S5

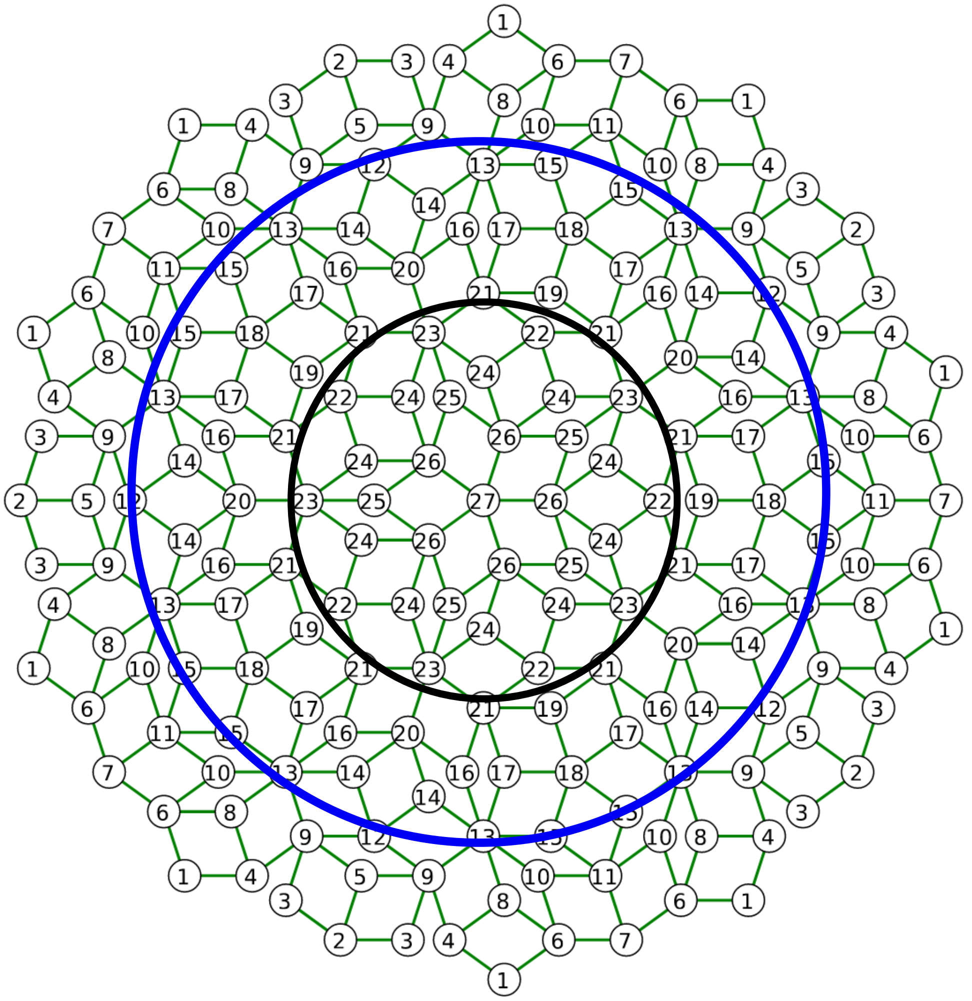

Figure S1: **Graph automorphism**. Graph automorphism orbits used to classify symmetry-related Penrose lattice sites. Sites automorphic to each other fall into one of the 27 equivalence classes indicated in the figure. Black and blue circles delimit the qubit classes included in the smaller lattices in Figs. S3 and S4.

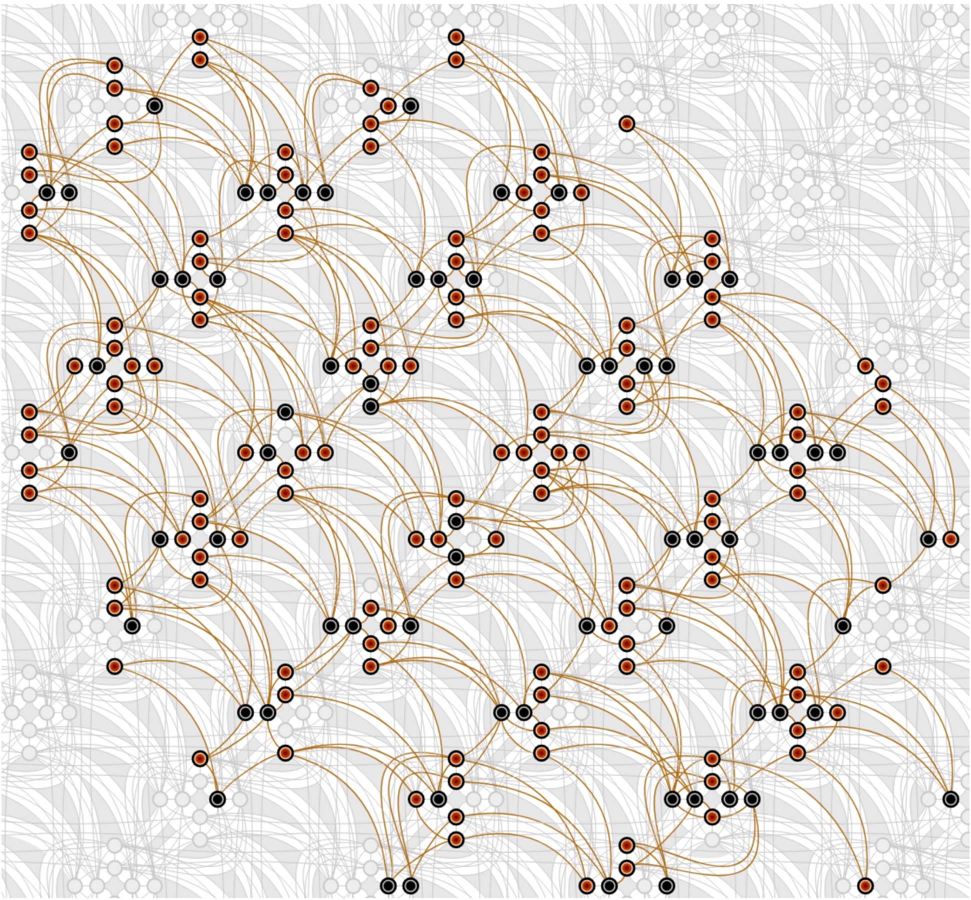

Figure S2: **201-qubit quasicrystal embedding**. Eight embeddings of similar connectivity using a single-qubit per quasicrystal site are simultaneously implemented on the Pegasus quantum processing unit.

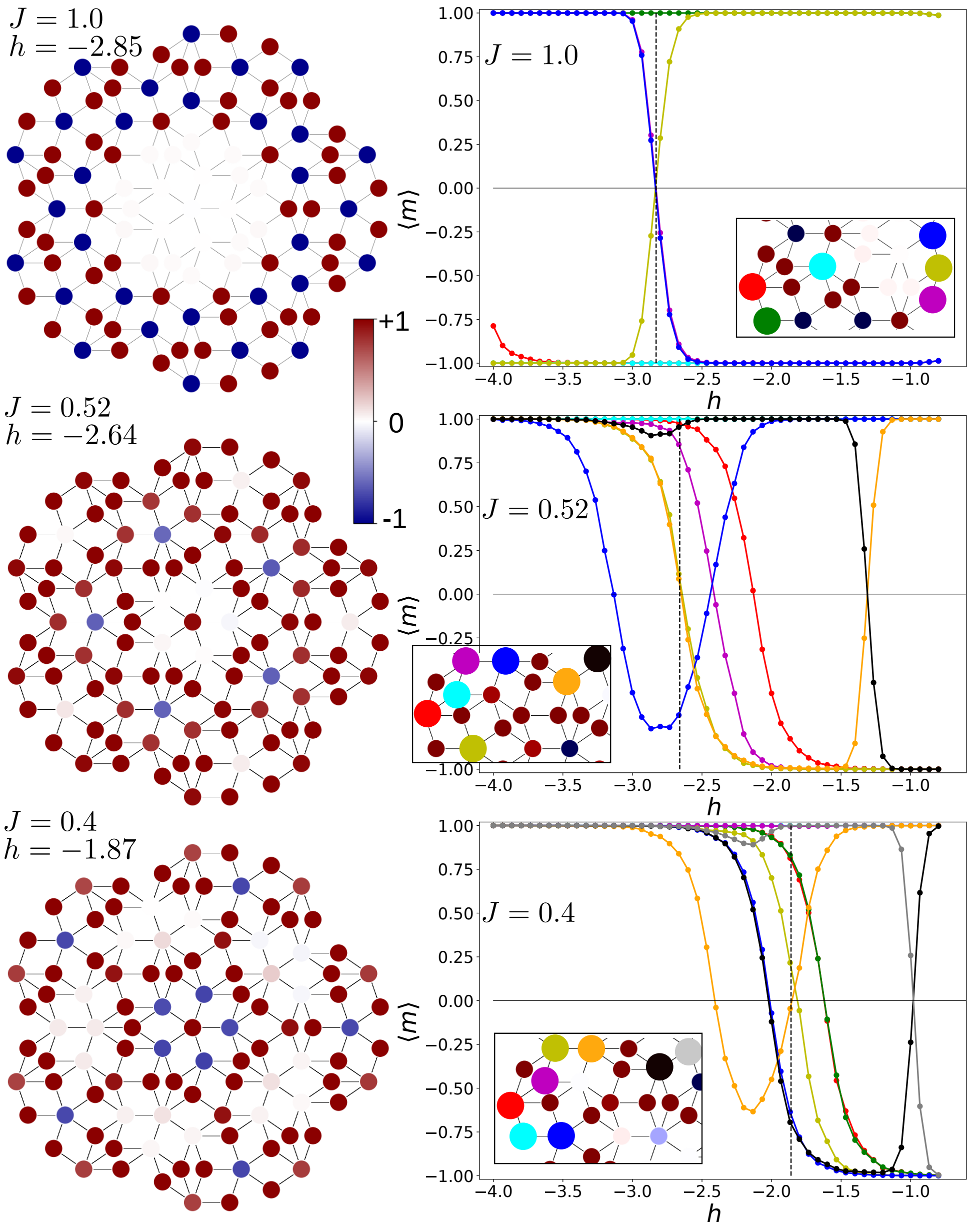

Figure S3: **Qubit-resolved averaged magnetizations of the 106-qubit quasicrystal.** Left panels: magnetic textures under several longitudinal fields of the 106-qubit Penrose quasicrystal studied in this paper. Evolution of $\langle m \rangle$ with applied longitudinal field for couplings $J = 1.0$, $J = 0.52$, and $J = 0.4$.

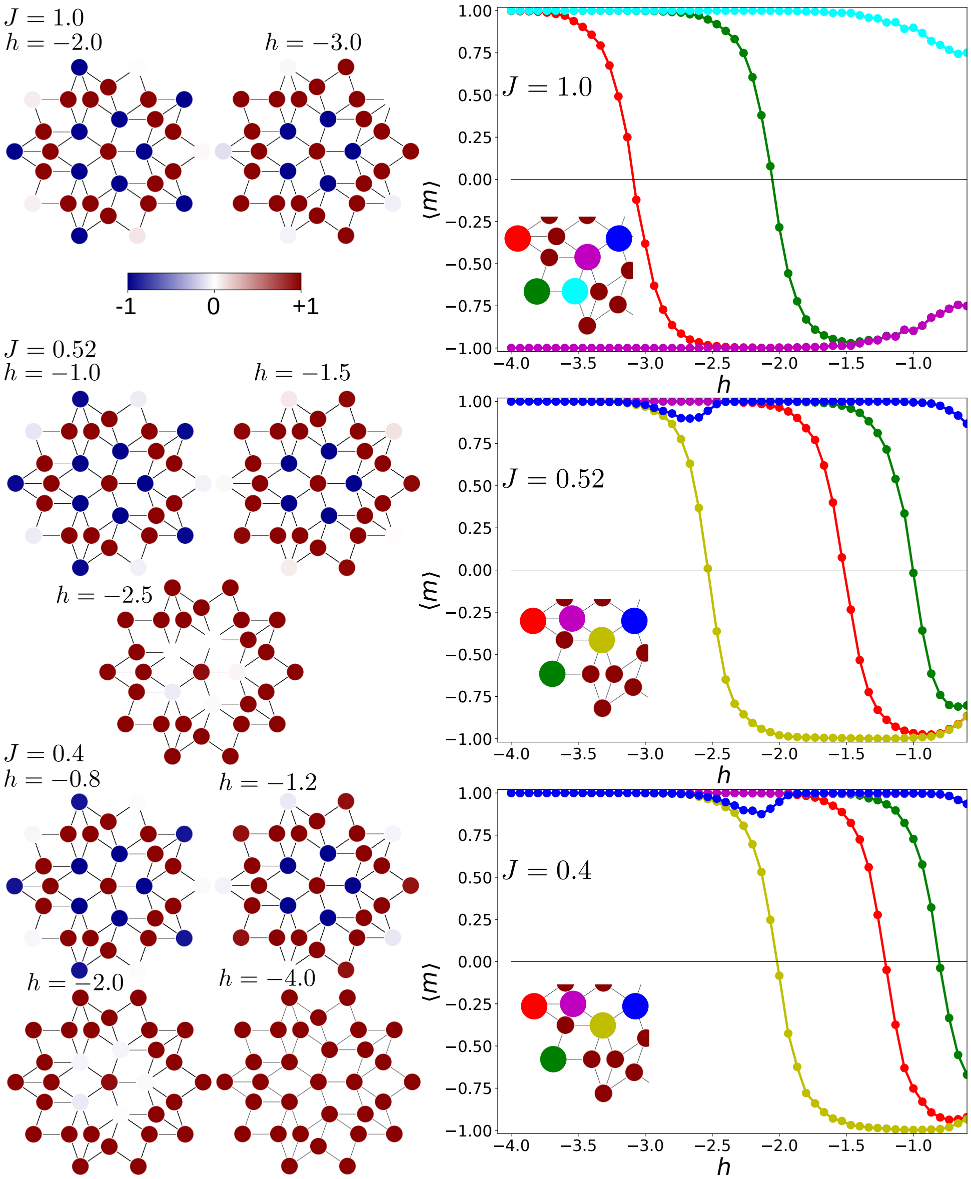

Figure S4: **Qubit-resolved averaged magnetizations of the 31-qubit quasicrystal.** Left panels: magnetic textures under several longitudinal fields of the central-region Penrose quasicrystal (31-qubits) studied in this paper. Evolution of $\langle m \rangle$ with applied longitudinal field for couplings $J = 1.0$, $J = 0.52$, and $J = 0.4$.

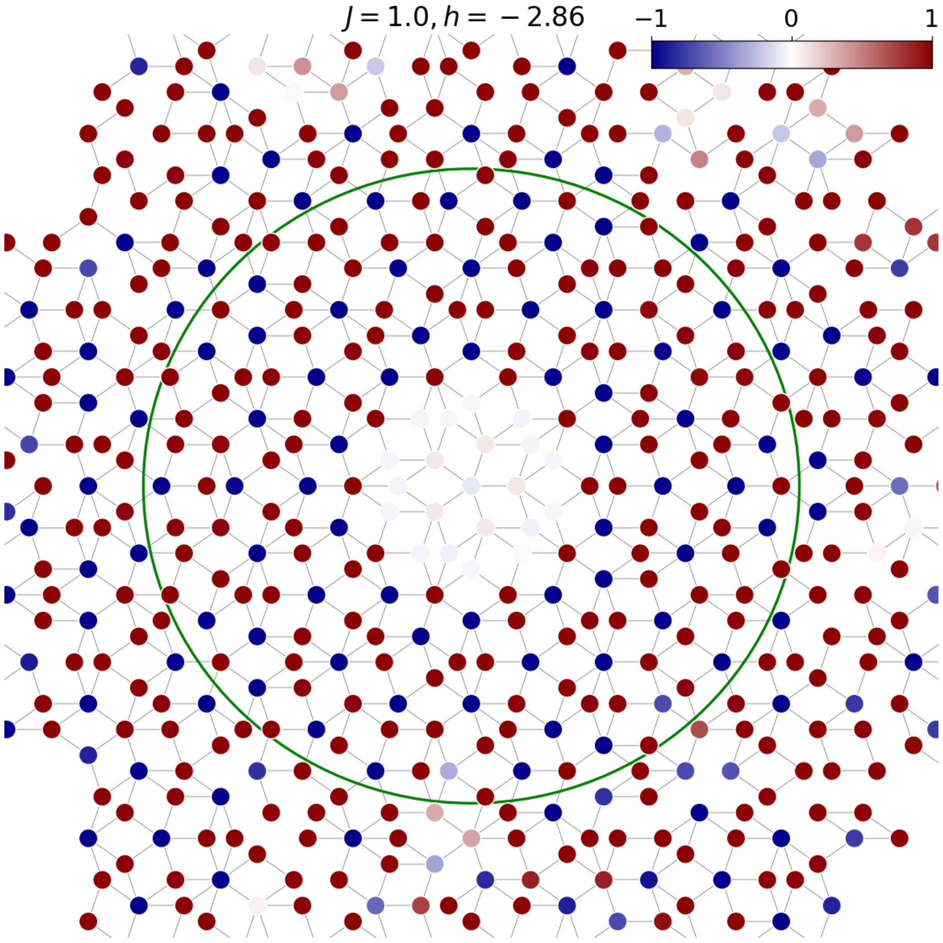

Figure S5: **Qubit-resolved averaged magnetization of the 600-qubit quasicrystal.** The central magnetic phase observed at $J = 1.0$ and $h \sim -2.86$ in two smaller quasicrystals is reproduced on the defect-rich 600-qubit lattice. Green line delimits the 201-qubit defect-free lattice.

Videos

File: Movie-S1.mp4

Evolution of the average magnetization of a 201-qubit quasicrystal. Animated sequence of the qubit-resolved average magnetizations (left panels), global average magnetization $\langle m \rangle$ (central panels), and structure factor $S(q)$ (right panels) at each applied longitudinal field h and coupling strength $J = 0.2$.

File: Movie-S2.mp4

Evolution of the average magnetization of a 201-qubit quasicrystal. Animated sequence of the qubit-resolved average magnetizations (left panels), global average magnetization $\langle m \rangle$ (central panels), and structure factor $S(q)$ (right panels) at each applied longitudinal field h and coupling strength $J = 0.3$.

File: Movie-S3.mp4

Evolution of the average magnetization of a 201-qubit quasicrystal. Animated sequence of the qubit-resolved average magnetizations (left panels), global average magnetization $\langle m \rangle$ (central panels), and structure factor $S(q)$ (right panels) at each applied longitudinal field h and coupling strength $J = 0.4$.

File: Movie-S4.mp4

Evolution of the average magnetization of a 201-qubit quasicrystal. Animated sequence of the qubit-resolved average magnetizations (left panels), global average magnetization $\langle m \rangle$ (central panels), and structure factor $S(q)$ (right panels) at each applied longitudinal field h and coupling strength $J = 0.5$. File: Movie-S5.mp4

Evolution of the average magnetization of a 201-qubit quasicrystal. Animated sequence of the qubit-resolved average magnetizations (left panels), global average magnetization $\langle m \rangle$ (central panels), and structure factor $S(q)$ (right panels) at each applied longitudinal field h and coupling strength $J = 0.6$.

File: Movie-S6.mp4

Evolution of the average magnetization of a 201-qubit quasicrystal. Animated sequence of the qubit-resolved average magnetizations (left panels), global average magnetization $\langle m \rangle$ (central panels), and structure factor $S(q)$ (right panels) at each applied longitudinal field h and coupling strength $J = 0.7$.

File: Movie-S7.mp4

Evolution of the average magnetization of a 201-qubit quasicrystal. Animated sequence of the qubit-resolved average magnetizations (left panels), global average magnetization $\langle m \rangle$ (central panels), and structure factor $S(q)$ (right panels) at each applied longitudinal field h and coupling strength $J = 0.8$.

File: Movie-S8.mp4

Evolution of the average magnetization of a 201-qubit quasicrystal. Animated sequence of the qubit-resolved average magnetizations (left panels), global average magnetization $\langle m \rangle$ (central panels), and structure factor $S(q)$ (right panels) at each applied longitudinal field h and coupling strength $J = 0.9$.

File: Movie-S9.mp4

Evolution of the average magnetization of a 201-qubit quasicrystal. Animated sequence of the qubit-resolved average magnetizations (left panels), global average magnetization $\langle m \rangle$ (central panels), and structure factor $S(q)$ (right panels) at each applied longitudinal field h and coupling strength $J = 1.0$.
